# Low-level toxicity of chemicals: No acceptable levels?

**DOI:** 10.1371/journal.pbio.2003066

**Published:** 2017-12-19

**Authors:** Bruce P. Lanphear

**Affiliations:** Faculty of Health Sciences, Simon Fraser University, Burnaby, Canada; National Institute of Environmental Health Sciences, United States of America

## Abstract

Over the past 3 decades, in a series of studies on some of the most extensively studied toxic chemicals and pollutants, scientists have found that the amount of toxic chemical linked with the development of a disease or death—which is central to determining "safe" or "hazardous" levels—is proportionately greater at the lowest dose or levels of exposure. These results, which are contrary to the way the United States Environmental Protection Agency (EPA) and other regulatory agencies assess the risk of chemicals, indicate that we have underestimated the impact of toxic chemicals on death and disease. If widely disseminated chemicals and pollutants—like radon, lead, airborne particles, asbestos, tobacco, and benzene—do not exhibit a threshold and are proportionately more toxic at the lowest levels of exposure, we will need to achieve near-zero exposures to protect public health.

This Perspective is part of the *Challenges in Environmental Health: Closing the Gap between Evidence and Regulations Collection*.

## Introduction

During medical school, I was taught to categorize patients with or without a disease. I learned that diseases were usually the result of exposure to a single agent and that toxic chemicals, like lead, exhibited a threshold; low concentrations of chemicals that we are regularly exposed to—which are now typically measured in parts per billions—were considered safe or innocuous. In my postdoctoral training, it became clear that most chronic diseases, like autism and heart disease, exist on a spectrum, and they usually result from the cumulative impact of many subtle risk factors. Over the past 15 years, I’ve also learned that exposure to exceedingly low concentrations of toxic chemicals, like lead, pesticides, and flame retardants, can be hazardous, especially if exposure occurs during early brain development; in many cases, there is no apparent threshold or safe level [1].

**Fig 1:**
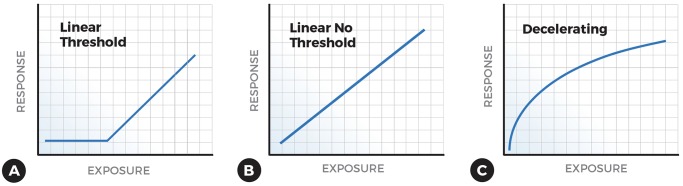
Examples of exposure response relationships: Linear threshold (A); linear, no threshold (B); and decelerating (C).

My appreciation for various dose-response or exposure-response curves has also grown (Fig 1). The first time I encountered a supralinear or decelerating dose-response curve in my own research, it didn’t register [2]. After we published our first 3 studies on the association of low-level blood lead concentration and children’s intellectual abilities [2–4]—which was in the shape of a decelerating curve in all 3 studies—I couldn’t stop thinking about it because it challenged the way we assess risk. A dose-response or exposure-response curve that is steeper at the lowest dose or levels of exposure is called a supralinear or decelerating curve (Fig 1C). Over the past 10 or more years, I’ve searched for other examples of toxic chemicals that exhibit a similar shape and tried to understand how they might change the way the government agencies regulate chemicals and, ultimately, prevent disease.

**Fig 2:**
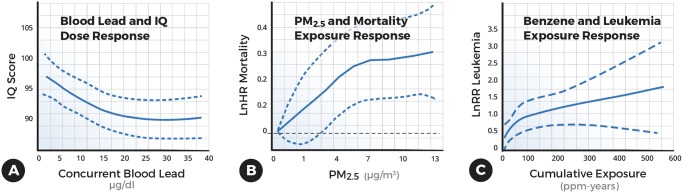
Examples of decelerating dose-response or exposure-response curves. (A) Blood lead concentration and intelligence quotient (IQ) scores reused from [4]; (B) fine particulate matter (PM_2.5_) matter and natural logarithm (Ln) relative risks (RRs) for nonaccidental mortality reused from [28]; and (C) benzene and natural logarithm (Ln) hazard ratios (HRs) for leukemia reused from [14].

Toxic chemicals often exhibit a decelerating dose-response or exposure-response curve (Fig 2). A decelerating response curve has been reported for ionizing radiation and lung cancer and leukemia [5–7], lead and intelligence quotient (IQ) scores (Fig 2A) [2–4, 8–11], fine airborne particles (particulate matter < 2.5 microns in diameter [PM_2.5_]) and mortality (Fig 2B) [12–13], benzene and leukemia (Fig 2C) [14], and asbestos and mesothelioma [15–16], as well as for tobacco and air pollution and birth weight or small-for-gestational age [17–19]. A decelerating dose-response or exposure-response curve has also been observed for arsenic and lung cancer [20], tobacco and reading deficits [21], and other occupationally induced diseases, but they have not been replicated [22–23].

Evidence that some toxic chemicals consistently exhibit a decelerating response challenges the central concepts of toxicology and risk assessment. For toxic chemicals that are not suspected of causing cancer, the EPA and other regulatory agencies assume that homeostatic and other repair mechanisms in the body result in a population threshold (Fig 1A); low doses or exposures are assumed to be inconsequential [24–25]. For carcinogens, the EPA assumes that the response is linear and without a threshold—what radiation scientists call the linear, no threshold (LNT) model (Fig 1B) [24–25]. These concepts, which are deeply entrenched and only occasionally questioned, were adopted before there was evidence to support them. In a series of studies conducted over the past 3 decades, investigators have found that neither of these assumptions is valid for radon, lead, fine airborne particles, asbestos, tobacco, and benzene and their associations with specific conditions or causes of death.

## Low-level ionizing radiation

In 1987, Rick Hornung and Ted Meinhardt, who were at the National Institute for Occupational Safety and Health (NIOSH), found that the association of radon with lung cancer resembled a decelerating exposure response curve [5]. John Gorfman, who was then a professor at the University of California, Berkeley, raised warnings about the risk of cancer from low-dose ionizing radiation, including evidence for proportionately elevated risk in the lower ranges of exposure [6]. Mark Little and his colleagues, who are at the Radiation Effects Research Foundation in Hiroshima, Japan, concluded that the shape of the exposure-response curve for leukemia among atomic bomb survivors was attenuated at higher levels [7]. In contrast, Klervi Leuraud and her colleagues, reported a linear increase in chronic myelogenous leukemia in their study of over 300,000 workers who were monitored for extremely low-level radiation exposures [26].

## Lead and intellectual abilities

Our earlier observations of a decelerating dose-response curve for childhood lead exposure and intellectual or academic abilities have been replicated in over a dozen studies [2–4, 8–11]. The impact of a decelerating relationship on lead-associated IQ deficits is striking: an increase in blood lead from <1 μg/dL to 30 μg/dL (<10 ppb to 300 ppb) was associated with a 9.2 IQ deficit, but the largest fraction of the deficit (6.2 IQ points) occurred below 10 μg/dL (100 ppb) (Fig 2A) [4]. David Bellinger, who is a professor at Harvard University and an expert in the neurotoxicity of environmental contaminants, estimated that, despite the dramatic decline in blood lead levels, lead exposure accounts for a loss of 23 million IQ points in a 6-year birth cohort of US children [27].

## Air pollutants and cardiovascular disease (CVD) mortality

Arden Pope, who is a professor of economics at Brigham Young University in Salt Lake City, carefully evaluated the exposure-response curve for fine airborne particles and cardiovascular mortality and showed it was decelerating [12]. Since then, other investigators have consistently observed a decelerating exposure-response curve for fine airborne particles and CVD mortality [13, 28]. In a large national study of Canadians, Pinault and coworkers found elevations in nonaccidental mortality down to levels of 1 μg/m_3_ (Fig 2B) [28]. A similar picture has emerged in cities around the world for CVD deaths. (See air pollution video)

Natural history studies of bans on smoking in public places have found surprisingly large reductions in heart attacks and preterm births [29–30]. In Scotland, for example, the ban led to a 20% reduction in heart attacks among nonsmoking adults and a 15% reduction in preterm births among nonsmoking pregnant women [31–32]. These reductions, which are as sizable as those achieved by many pharmacologic agents, provide further evidence that exceedingly low-level exposures to airborne particles or secondhand smoke substantially contribute to death and disability.

## Benzene and leukemia

Jelle Vlaanderen and Roel Vermeulen, who are at Utrecht University in the Netherlands, along with an international team of scientists, pooled data from 9 cohort and case-control studies of benzene-exposed workers. A decelerating exposure-response curve fit their data better than a linear one (Fig 2C); they observed relative risks of 1.52 (95% CI 1.08–2.15), 1.73 (95% CI 1.27–2.34), and 2.11 (95% CI 1.51–2.96) for cumulative benzene exposures of 10, 20, and 40 ppm-years, respectively (Fig 2C) [14].

## Asbestos and malignant mesothelioma

Asbestos production and use has declined in affluent countries over the past several decades. Still, because of its long latency period, deaths from mesothelioma are only now peaking in North America and Europe; deaths from mesothelioma will continue to rise in industrializing countries that allow the use of asbestos. Hodgson and Dartnon conducted a meta-analysis of asbestos and mesothelioma and found that the shape of the exposure-response resembled a decelerating curve [15]. Wayne Berman and Kenny Crump, who conducted a reanalysis of 5 cohorts of asbestos-exposed workers, observed a decelerating exposure-response curve in every cohort they studied [16].

## Mechanism for the decelerating response curve

What is the mechanism for the decelerating response curve? It may be due to a biologic effect. For example, there is an exposure-related production of the toxic metabolites of benzene: muconic acid and hydroquinone production is attenuated at higher exposures to benzene [33–34]. The underlying mechanism that produces the decelerating shape is less apparent for the other toxic chemicals. Unmeasured confounders could bias or distort the shape of the dose-response or exposure-response relationship, but it is unlikely that so many studies from different populations would all be subject to the same type of bias [35]. Moreover, if confounding does explain the decelerating response, it is more likely to explain the attenuated relationship observed at higher exposures because many of the recognized confounders are more common among populations that are more heavily exposed to chemicals [35].

Industry-funded critics have argued that the decelerating dose-response or exposure-response represents a statistical artifact, but there is no evidence for this assertion [35–36]. Poorer measurement of exposures at higher concentrations (i.e., exposure misclassification) is another possible explanation, but this is unlikely at the ranges of exposure studied. It is likely, however, that many studies did not identify a decelerating response curve because they did not test for it or the range of exposure was too limited. In a pooled study of childhood lead exposure, for example, we found that the dose-response curve was decelerating in the larger sample, which had blood lead concentrations up to 30 μg/dL (300 ppb), but it was linear for the smaller subset of children who had blood lead <7.5 μg/dL (<75 ppb) [4]. Other possible reasons include the healthy worker effect and depletion of susceptible persons [22].

## Residual questions and quandaries

These studies indicate, for a given level of exposure, that there are proportionately greater harms or steeper increases in risk at lower levels of exposure for some of the most extensively studied toxic chemicals and pollutants. Indeed, this array of studies indicates that epidemiologists, toxicologists, and risk assessors have markedly underestimated the contribution of toxic chemicals to the development of prevalent chronic diseases, including CVD, mesothelioma, leukemia, and learning problems. Some scientists will undoubtedly argue that it is necessary to identify the underlying mechanism of toxicity before the EPA or other regulatory agencies promulgate stricter environmental health standards. It is useful to know the mechanism, and we should invest in studies to identify them, but it is not essential [37]. Indeed, it is often difficult to isolate a single specific mechanism of toxicity because many toxic chemicals are systemic toxicants.

These studies raise several intriguing questions and quandaries. For some toxic chemicals, like lead, it is plausible that chronic, low-level exposures could be toxic because our single-celled ancestors were exposed to exceedingly low concentrations of them in primordial waters and humans did not develop tolerance. But why haven’t humans developed mechanisms to tolerate exceedingly low-level exposures to toxic chemicals we evolved with, like particles generated from combustion? Clearly, further research is necessary to clarify the role of exposure assessment in the decelerating response curve, elucidate underlying mechanisms, and verify which toxic chemicals exhibit a decelerating response. A scientific committee should be convened to review the evidence, quantify the added impact on population health, and make recommendations about further research and how regulatory agencies should modify risk assessment.

## Implications for public health

Over the past century, as exposures to toxic chemicals have expanded beyond the workplace, the number of people exposed has increased dramatically, even if at lower levels of exposure. For toxic chemicals without a threshold—and especially for those that exhibit a decelerating shape—we will inevitably fail to prevent most deaths, diseases, and disabilities, like obesity, heart disease, diabetes, and cancer, until we expand our focus to include population strategies that target people who have low-to-moderate exposures. For example, if we limit our efforts by exclusively protecting the 500,000 (approximately 2.5%) US children who have a blood lead concentration higher than the current action level, which is set at 5 μg/dL (50 ppb), we would only preserve 3 million (18%) of the 23 million IQ points lost in a 6-year birth cohort [1].

The risk of developing a disease or dying is obviously higher for more heavily exposed populations, but the larger number of people who have low-to-moderate exposures and will ultimately develop a disease overwhelms the smaller number of cases among people who are more heavily exposed [38]. This concept, which is called the prevention paradox, is not universal: it does not apply if there is a threshold and only a small fraction of the population is exposed to higher levels of a toxic chemical or if most people are defined as high risk. (See prevention paradox video) Still, in the latter case, we would prescribe a population strategy. Moreover, unless we implement population strategies, people in the low-to-moderate exposure group will continually replenish the high-risk group. Finally, in a high-risk or clinical strategy, physicians typically wait for signs or symptoms of a disease to develop in a patient before they intervene.

The pattern of toxicity observed for these ubiquitous chemicals questions the basic assumptions about how agencies regulate chemicals and raises 2 distinct but related issues. First, consistent with assumptions we make about carcinogens, no threshold appears to exist for some ubiquitous, noncarcinogens. If so, this raises fundamental questions about how we conduct risk assessment and regulate noncarcinogenic chemicals. This is consistent with the National Academy of Science report “Advancing Risk Assessment,” which concluded that the US EPA should assume that there is a safe level of exposure for noncarcinogens only if there is strong evidence for a threshold [25]. Second, the steep increase in risk at the lowest levels followed by the flattening or attenuation at higher doses or levels of dose or exposure for carcinogenic and noncarcinogenic toxicants will challenge regulatory agencies to promulgate substantially larger reductions in exposures to toxic chemicals; incremental reductions, which have typically been promulgated in the past, are not sufficient to protect human health.

The policy implications of these studies are staggering. In theory, they indicate that regulatory agencies should strive to achieve near-zero exposures for radon, lead, airborne particles, asbestos, and benzene to protect people’s health. Regulating criteria pollutants that fall under the Clean Air Act—which includes lead and PM_2.5_—would be especially onerous because the US EPA administrator is required to “protect public health with an adequate margin of safety.”

Regulatory agencies have 2 often-conflicting goals about widespread exposures to toxic chemicals: to protect the health of the public and to make the public feel protected. If a regulatory agency suddenly declared that millions more people are at risk for death or disease from ubiquitous exposures to toxic chemicals, it would not be popular; exposures to many of these chemicals are widespread, and it would be difficult to rapidly ban or eliminate them. Unfortunately, even though population-level interventions to prevent disease and disability are more likely to be cost-beneficial or cost-effective than those that target 1 person at a time [39], they are difficult to implement in a health system dominated by the free market because the benefits of prevention are hard to privatize. It won’t be easy, but regulations to reduce widespread exposures to toxic chemicals will ultimately be enacted because the hazards are too great and the benefits too large.

## Abbreviations

CVD: cardiovascular disease
EPA: Environmental Protection Agency
IQ: intelligence quotient
LNT: linear, no threshold
NIOSH: National Institute for Occupational Safety and Health
PM_2.5_: particulate matter < 2.5 microns in diameter
